# Growing Pigs’ Interest in Enrichment Objects with Different Characteristics and Cleanliness

**DOI:** 10.3390/ani9030085

**Published:** 2019-03-08

**Authors:** Jean-Michel Beaudoin, Renée Bergeron, Nicolas Devillers, Jean-Paul Laforest

**Affiliations:** 1 Département des sciences animales, Université Laval, 2425 rue de l’Agriculture, Québec City, QC G1V 0A6, Canada; jean-paul.laforest@vrrh.ulaval.ca; 2 Department of Animal Biosciences, Animal Science and Nutrition, 50 Stone Road East, Guelph, ON N1G 2W1, Canada; rbergero@uoguelph.ca; 3 Agriculture and Agri-Food Canada, Sherbrooke Research and Development Centre, 2000 College Street, Sherbrooke, QC J1M 0C8, Canada; nicolas.devillers@canada.ca

**Keywords:** fattening pigs, pig behavior, animal welfare, environmental enrichment

## Abstract

**Simple Summary:**

The modern swine industry is mostly based on an intensive production model that has evolved under economic pressure and has shaped rearing facilities around production optimization rather than the natural needs of pigs. Barren rearing spaces for growing pigs do not allow them to fully express their natural behaviors (e.g., rooting and chewing). This can lead to the emergence of abnormal behaviors, such as tail-biting which causes stress to the animals, and potential financial loss. A simple strategy to allow pigs to express their natural behaviors is to add enrichment objects to the rearing environment. However, pigs tend to lose interest in the objects rapidly. The characteristics of an object, such as the degree of cleanliness and malleability of the material used, can significantly increase its attractiveness. This study first compared seven different objects based on the level of manipulation received from growing pigs. A block of dried wood that was presented on the floor had the longest manipulation time. Secondly, four objects were compared for their level of cleanliness or wear and no differences in manipulation were found between objects that were cleaned or replaced daily and objects that were not cleaned or replaced (for a period of five days).

**Abstract:**

Enrichment objects can be a practical way to provide rooting and chewing material to growing pigs, on which they can express species-specific behaviors. The challenge is to provide enrichment objects that will satisfy pigs’ behavioral needs, while being practical and low-cost for the producers. Two trials were conducted to evaluate the effects of object characteristics such as design, location, cleanliness or degree of wear, on pigs’ interest over time. The first trial compared seven objects, varying in their design and location, presented individually for five consecutive days to groups of 12 ± 3 (average ± SD) pigs, weighing 61 ± 9.2 kg. The pigs’ interest in the objects was evaluated based on the frequency, total duration and mean length of manipulation with the objects. All objects were manipulated at different levels depending on their characteristics. On average, the pigs interacted more frequently (*p* < 0.001) with a chewable object made of three polyurethane balls, spring-mounted and anchored to the floor, and spent more time manipulating a dried wood beam on the floor (*p* < 0.05), which was destructible and chewable, than suspended ropes, plastics and rubber objects, and a plastic ball on the floor. The second trial used two-choice preference tests to compare objects varying in their degree of cleanliness or wear, presented in pairs to growing pigs weighing 47 ± 7 kg and housed in groups of 14 ± 1. Two identical objects were placed simultaneously in a pen over 5 days, and only one of them was cleaned or replaced daily (treatment) while the duplicate was left untouched (control). The results showed no clear preference between control and treatment objects, indicating that short-term maintenance of the objects might be unnecessary.

## 1. Introduction

The welfare of growing pigs has been an increasing concern over recent years and is becoming an important pillar to ensure the sustainability of the swine industry [1]. In many swine-producing countries, pigs are typically raised in barren pens without litter [2]. Such an environment does not allow pigs to fully express their natural species-specific behaviors, which can lead to psychological distress [3]. These pigs become more likely to express abnormal behaviors, such as tail-biting, to cope with the repression of their natural behaviors [3] or as a result of frustration [4]. Tail-biting between pigs can affect health, growth and the welfare of victim pigs and cause significant economic losses for producers [2,5,6]. In European countries, it was estimated that 30% to 70% of farms had tail-biting problems to some extent, with 1% to 5% of pigs presenting tails with lesions [2].

Negative impacts of barren pens can be reduced by providing enrichment objects to the pigs, on which they can express some species-specific behaviors [3,7]. Other well-studied solutions include the provision of straw or other similar rootable materials, which have been shown to reduce aggression and enhance welfare (e.g., Moinard et al. [8]). However, the slurry system of fully or partly slatted pens is at risk of being blocked by straw or similar substrates [3,9]. Therefore, it is necessary to find alternative solutions such as enrichment objects. However, pigs’ interest in the objects is likely to be influenced by the type of material and positioning within the pen [10,11]. Chewable and destructible materials, such as wood and rubber, are more likely to sustain interest compared to harder materials (e.g., metal chains) [10]. On the other hand, objects that are suspended at shoulder level to the pigs in the pen could initiate more manipulation since they are more visible and they stay cleaner than objects on the floor [7]. Enrichment objects associated with high levels of interest are more likely to reduce injurious social behaviors between pigs [3,11]. Therefore, tail- and ear-biting may be reduced by finding the most appropriate objects or combination of objects (e.g., Telkänranta et al. [12]). However, interest in an object can decrease after a few days [13,14] or a few weeks [15]. To prevent a rapid loss of interest, it is important to know the frequency at which objects should be replaced.

Additionally, soiled objects (by feces or dirt) potentially decrease pigs’ interest [16] compared to clean objects, reducing their enrichment value. In a similar way, some highly destructible objects, such as ropes, can lose some of their attractiveness after a while because they are rapidly destroyed [12,17]. Although destructibility is seen as a beneficial characteristic for an enrichment object [10], it increases the frequency of object replacement. To be used on farms, enrichment objects should be practical and improve the economics of production [3]. Therefore, it is important to know whether washing or renewing objects is necessary to maintain their enrichment value.

This study was divided into two trials aimed at gathering information about the attractiveness of multiple enrichment objects presented to growing pigs. The objective of the first trial was to evaluate the short-term attractiveness of seven different objects based solely on their characteristics. The hypothesis was that destructible and chewable objects would trigger more manipulations. The objective of the second trial was to evaluate the effect of an object being soiled or damaged over time on its attractiveness. The hypothesis was that a clean or new object would be more attractive.

## 2. Materials and Methods

### 2.1. Animals and Housing

Large White × Landrace (or reciprocal mating) barrows were provided by one of the seven members of PigGen Canada from healthy multiplier farms and were group-housed in fully slatted pens measuring 2.6 m × 4.9 m in a research facility in Deschambault, Canada. Lights were on from 08:00 to 16:00 and no outside light could reach the pens. For the first trial, the average number of pigs per pen was 12 ± 3 (± SD), weighing 61 ± 9 kg on average, at the midpoint of the five-day recording periods. In total, 28 pens were used, representing 328 pigs. Due to a limited number of available pens containing pigs within the required weight range, eight pens were used twice, but never in a row. The weight range was 30 kg for trial one, meaning that all pens’ average weight had to be between 45 kg and 75 kg during observations. The first trial lasted from May to August. For trial two, the average number of pigs per pen was 13.5 ± 1, weighing 47 ± 7 kg on average, at the midpoint of the five-day recording periods. Eight different pens and their pigs were also used twice because of a limited availability of pigs, but not for two consecutive observation periods, representing 107 different pigs. The weight range was 20 kg, meaning that all pens’ average weight had to be between 37 kg to 57 kg during observations. The second trial lasted from November to December. For both trials, animals were fed a pelleted (corn- and soya-based) commercial diet and had ad libitum access to food and water. Two suspended metal chains were available in each pen before the beginning of the experiments and were removed during the trials. All animals were handled and cared for according to the guidelines of the Canadian Council on Animal Care [18], and the animal care committee at Laval University approved the procedures (CPAUL, protocol number 2015091).

### 2.2. Enrichment Objects

**Figure 1:**
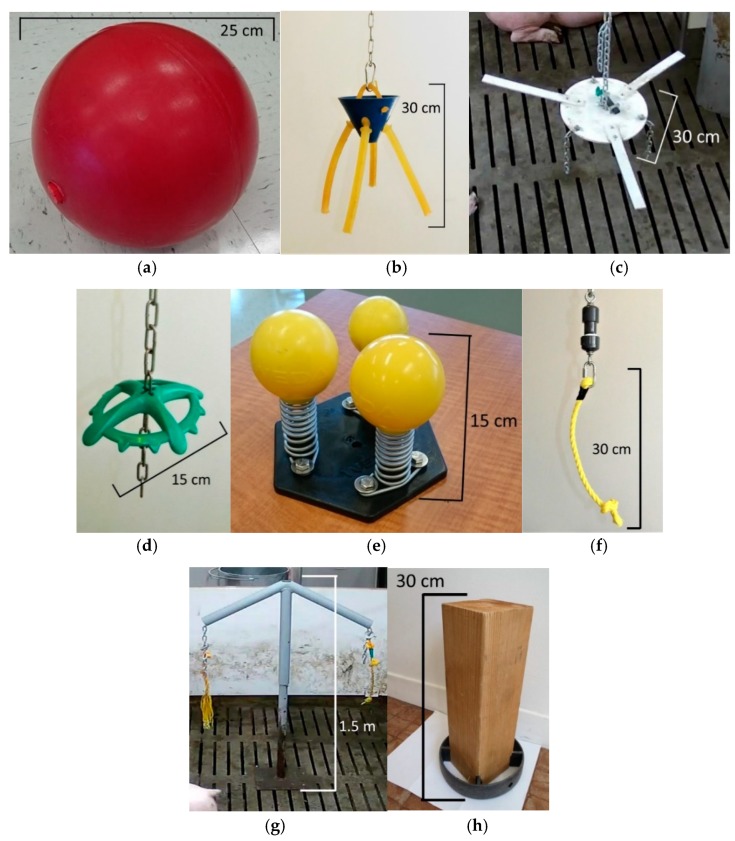
Enrichment objects presented to growing pigs for five days in either one or both trials: (**a**) Ball; (**b**) Bite-Rite; (**c**) Disc; (**d**) Porcichew; (**e**) Rooting Cones; (**f**) Rope; (**g**) Seesaw; (**h**) Wood.

All objects (shown in Figure 1) were selected to cover many of the material characteristics (e.g., deformable, destructible, chewable, odorous) and locations in the pen (e.g., suspended, free in the pen, fixed on the floor) that have been reported to impact their degree of attractiveness [13]. Some objects were commercially available, whereas others were custom-made, as indicated in the list below:

- Ball: A commercial ball (Boomer Ball, Grayslake, IL, USA) made of rigid plastic and measuring 25 cm in diameter. The ball was loose in the pen and the pigs could play with it freely.
- Bite-Rite: A commercial object (Ikadan System A/S, Ikast, Denmark) suspended from the ceiling and comprising four chew sticks made of rubber-like material (2.2 cm in diameter, 25 cm in length) fixed to a central plastic cone. The chew sticks were at shoulder level to the pigs.
- Disc: A custom-made (Dundalk Plastic-Fab, Horning’s Mills, ON, Canada) plastic disc (30 cm in diameter) suspended from the ceiling at shoulder level to the pigs. It had three hanging chains (15 cm in length) and three plastic strips (30 cm × 3 cm) fixed around it.
- Porcichew: A commercial object (Ketchum Manufacturing Ltd., Brockville, ON, Canada) made of a chewable plastic ring (15 cm in diameter) suspended from the ceiling at shoulder level to the pigs. The ring had a green apple scent.
- Rooting Cones: A commercial object (WEDA Dammann and Westerkamp GmbH, Goldenstedt, Germany) consisting of three chewable polyurethane balls (2 × 8 cm and 1 × 6 cm in diameter) fixed on top of metal springs (7 cm in length), which were mounted on a plastic ground plate anchored to the floor of the pen.
- Rope: A 30-cm-long polypropylene rope (Everbilt, Atlanta, GA, USA) suspended from the ceiling with a knot at the free end, which was at shoulder level to the pigs.
- Seesaw: A custom-made object (Agriculture and Agri-Food Canada, Sherbrooke Research and Development Centre, Sherbrooke, QC, Canada) consisting of two metal tubes forming a “T” (1.5 m in height, 1.2 m in width), which was fixed to the pen floor. Two polypropylene ropes (30 cm in length, 1.3 cm in diameter) were attached at both ends of a chain, which could slide inside the tilted tubes. The chain could slide for about 30 cm when a pig was pulling it from one side or the other. The tilted tubes could pivot 360° around the central axis. The ropes were presented at shoulder height of the pigs and had a knot at the free end to slow their destruction.
- Wood: A beam of untreated red cedar (10 cm × 10 cm × 30 cm) with a plastic ring (Jupiter Agro-Biotech, Saint-Hyacinthe, QC, Canada) on one end (4 cm in height, 25 cm in diameter) to elevate it from the floor for easier manipulations by the pigs and to reduce soiling. It was attached to a 1.5-m-long chain to limit its movements because the pigs could carry it to the feeders and block them.

### 2.3. Experimental Procedures

The first trial was conducted in a fully randomized experimental design, repeating every treatment four times. Seven treatments were used, representing the seven enrichment objects (Ball, Bite-Rite, Disc, Porcichew, Rooting Cones, Seesaw, and Wood, as shown in Figure 1). Each week, four different objects were individually placed in four pens for five consecutive days. The objects were presented the afternoon before the recording started (day 0) to make sure that all the pigs were accustomed to the objects the next morning. Behavioral recordings were done on days 1 to 5 and on day 6, the objects were removed and cleaned. A group of pigs could not be used two weeks in a row for all eight of the pens that were used twice. Objects on the floor were placed in the cleanest part of the pen, far enough from the walls to allow all-round access. Suspended objects were also presented where the pigs could manipulate them from all sides. These locations were kept as similar as possible between weeks and pens.

The second trial was conducted in a complete block design and the effect of cleaning or replacing four selected objects was evaluated through a preference test, which was repeated four times. Two identical objects were presented at the same time for five consecutive days in one of four barren pens. One randomly selected object was not cleaned or replaced for five days (control object), whereas its duplicate was cleaned or replaced every morning from day 1 to 5 (treatment object). Treatment and control objects remained the same during those five days. As in trial one, the locations of every object were kept as similar as possible between repetitions.

To evaluate the effect of cleanliness, three commercial objects (Ball, Bite-Rite and Rooting Cones) were selected based on their characteristics and on information gathered in the first trial. The Ball and the Rooting Cones were shown to be rapidly soiled with feces and dirt as they were manipulated on the floor. In contrast, the chewing sticks on the Bite-Rite were only moderately soiled. Cleaning was performed using water and paper towels to remove dirt and feces on the Ball, chewing sticks of the Bite-Rite and three polyurethane balls, springs and ground plate of the Rooting Cones. No soap was used to avoid affecting the smell of the objects. Afterwards, objects were dried with paper towels. To determine the effect of object replacement, a suspended rope (shown in Figure 1) was chosen because of its rapidly destructible nature [17]. The treatment Rope was replaced with a new and identical rope daily.

During the treatment procedure (replacement or cleaning), both objects (control and treated) were taken outside the pen and were put back in at the same time, except for the Rooting Cones, because they could not be removed easily from the pen. Cleaning of the Rooting Cones was done in the pen, and the control Rooting Cones were also manipulated for the same amount of time, simulating a cleaning process. These procedures were performed to control for the potential effects of manipulation by employees on the degree of attractiveness of experimental objects.

### 2.4. Behavioral Observations

Behaviors were video recorded for both trials with cameras (TRENDnet: model TV-IP310PI, Torrance, CA, USA) fixed to the ceiling, providing a complete view around the objects. Recording was performed from 08:30 to 16:30 (when lights were on in the barn). A preliminary trial showed that pigs did not manipulate the objects much during darkness periods. Behavioral observation was performed by video analysis and focused on behaviors called manipulations, which were defined as any object-directed behaviors performed intentionally, involving a contact with the snout, head, legs (push, hit, rub) or with the mouth (chew, bite, pull, shake). Two variables were gathered directly from video analyses: total time spent manipulating the objects, calculated in terms of duration (rounded to the nearest second), and the frequency of manipulation from day 1 to 5. A third variable was the mean manipulation length and was obtained from the division of the duration by the frequency. Two manipulations were considered distinct when separated by a minimum of four seconds with no object-directed behavior performed by any pig (similar to the five seconds proposed by Gifford et al. [19]). A manipulation could be performed by multiple pigs simultaneously and it did not matter if the initial pig in contact with the object was not the same as the last one.

Behavioral sampling occurred over four continuous time periods of one hour each, equally distributed throughout the light period (09:00–10:00, 11:00–12:00, 13:00–14:00, 15:00–16:00) for the first trial, and three continuous time periods of half an hour each (09:30–10:00, 11:30–12:00, 13:30–14:00) for the second trial. The number and length of the time periods were respectively reduced and shortened for the second trial based on the results of the first trial, suggesting that reliable data could be collected in this way.

### 2.5. Statistical Analysis

Data analysis was performed with the MIXED procedure of SAS 9.4 (SAS Institute, Cary, NC, USA), using repeated measures. Pens were set as experimental units and the variables analyzed were the mean duration, mean frequency and mean manipulation length per time period. For the first trial, data on the object, day of observation, and the interaction between day and object, were analyzed as fixed effects and the repetitions as random effects. Multiple comparisons between objects were performed using Tukey’s adjustments of Student’s *t*-test. All data were tested for normal distribution.

For the second trial, preferences were evaluated using delta values between the same two objects (differences in duration, frequency and mean manipulation length, separately), calculated for every pen on each day (1 to 5). A delta different from zero (Student’s *t*-test on each day) was interpreted as a preference. Manipulations of objects by the pigs on day 1 and each of the other days (2 to 5) were compared using Dunnett’s correction for all objects independently, with control and treated data combined. Data on mean manipulation length in trial one and on the manipulation duration for the Bite-Rite in trial two had to be subjected to a logarithmic and a square root transformation, respectively, to ensure a normal distribution before the repeated measures analyses. Data are presented as least square means ± SEM, unless otherwise stated.

## 3. Results

### 3.1. Trial One

**Figure 2:**
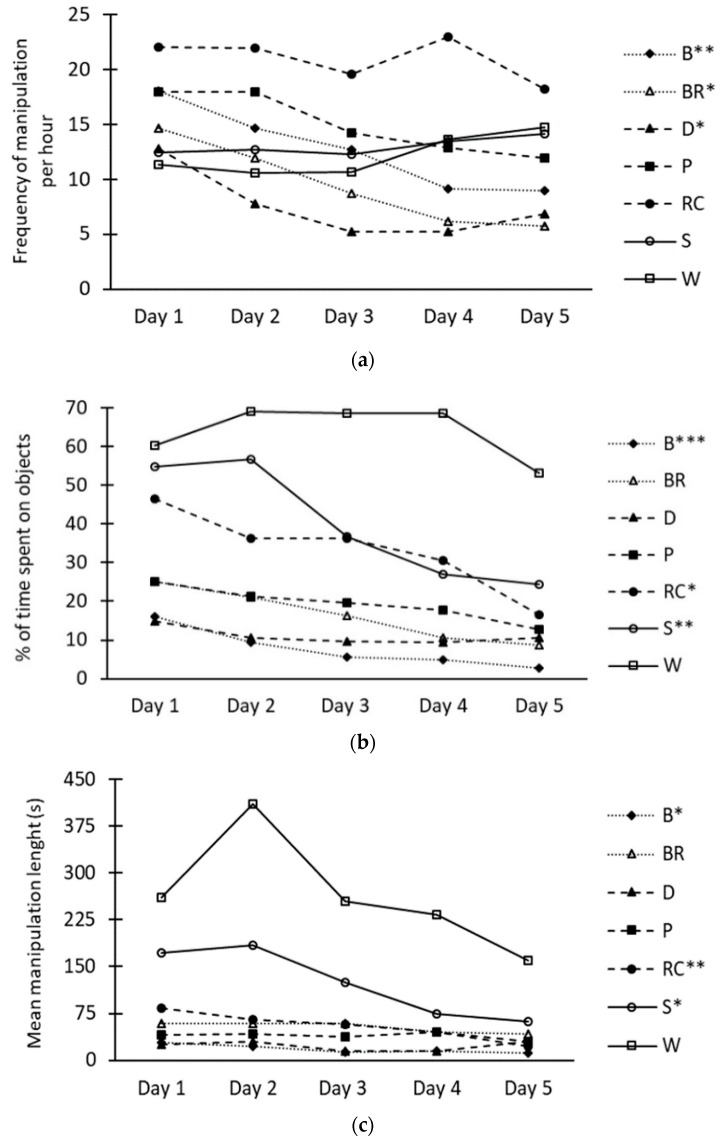
Daily adjusted means of the frequency of manipulation per hour (**a**), the proportion of time spent on objects (**b**) and the mean object manipulation length (**c**) in growing pigs. Objects (B: Ball, BR: Bite-Rite, D: Disc, P: Porcichew, RC: Rooting Cones, S: Seesaw and W: Wood) were presented individually in pens for five consecutive days. Significant effect of days is presented for each object separately with * (*p* < 0.05), ** (*p* < 0.01), *** (*p* < 0.001).

The frequency of manipulations (Figure 2a) was affected by object (*p* < 0.0001) and day (*p* < 0.001), and the interaction between object and day tended to be significant (*p* = 0.07). The Rooting Cones were the most frequently used objects by the pigs (21.0 times per hour on average), and the frequency of object manipulation was significantly reduced over time for the Ball, the Bite-Rite and the Disc between day 1 and day 5 (Figure 2a). There was an object–day interaction (*p* < 0.05) for the time spent manipulating objects (Figure 2b). The manipulation durations were consistently highest with Wood throughout the recording periods, while the percentage of time manipulating the Ball and the Rooting Cones decreased between day 1 and day 5 and between day 2 and day 5 for the Seesaw. An object–day interaction (*p* < 0.05) was also observed for mean manipulation length. The highest mean manipulation length was observed with Wood; it remained high over the five observation days, did not decrease significantly between day 1 and day 5 and remained higher than all other objects every day. The manipulation length with the Seesaw was higher on day 1 compared to day 5 (Figure 2c). The same has been found for the Ball and The Rooting Cones although they had lower manipulation lengths from day 1 compared with the Seesaw.

### 3.2. Trial Two

The pigs did not show any clear preference for control or treated (cleaned or replaced) objects. The only significant differences found were for the frequency of Ball manipulation on day 2 and the mean manipulation length of the Rooting Cones on day 3 (Table 1). Both indicated a preference for the control object and neither of these preferences persisted over time. The average frequency of manipulation for control and treated objects pooled together throughout the five experimental days were 19.2 ± 3.3 (Rope), 18.9 ± 1.5 (Rooting Cones), 16.7 ± 0.7 (Ball) and 9.9 ± 1.7 (Bite-Rite) manipulations per time period (30 min). The average percentages of time spent manipulating control and treated objects pooled together throughout the five experimental days were 32.4 ± 3.4 (Rope), 25.5 ± 1.8 (Rooting Cones), 16.7 ± 0.7 (Ball) and 13.6 ± 2.6 (Bite-Rite). Based on the frequency and duration data, the mean manipulation lengths in seconds were 45.8 ± 13.6 (Rope), 24.5 ± 8.9 (Bite-Rite), 23.2 ± 8.1 (Rooting Cones) and 15.2 ± 1.9 (Ball).

**Figure 3:**
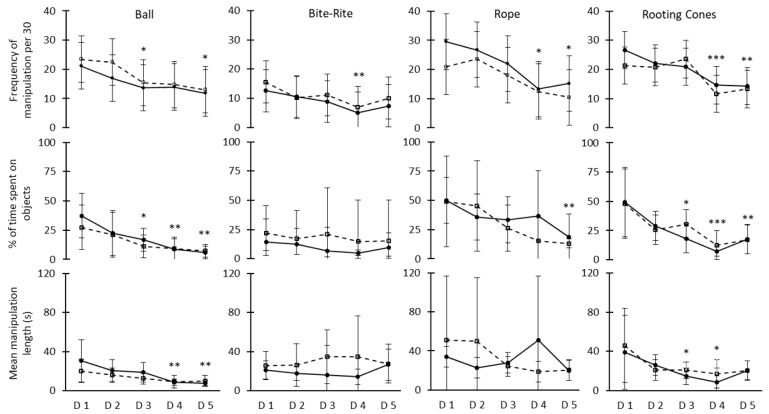
Adjusted means with 95% confidence intervals of frequency of manipulation per 30 min (upper row), percentage of time spent on manipulation (middle row) and mean manipulation length in seconds (lower row) for treated (solid line) and control (dash line) objects. Significant differences between daily average values (day 2 to day 5) from treated and control objects combined and the average value on day 1 (Dunnett’s correction) is shown with * (*p* < 0.05), ** (*p* < 0.01), *** (*p* < 0.001).

The four objects differed in their duration, frequency and mean length of manipulation over time (Figure 3). Interest was maintained at different relative levels between objects. The decrease in interest over time was observed between day 3 and day 5 and mainly affected the Ball and the Rooting Cones (Figure 3). Since no preferences were detected between control and treated objects (minus the two exceptions stated before), the pigs’ interest in control and treated objects was considered to remain similar over the five-day periods.

## 4. Discussion

### 4.1. Behavioral Analysis—Trial One

One of the main findings of the present study was that more time was spent manipulating the Wood compared to any other object presented to the pigs. This difference was observed on every experimental day and the pigs’ interest in the Wood did not decrease over the five-day period. Trickett et al. [17] have also reported such a sustained interest over time in dried wood. The wood beam has some of the key characteristics (e.g., destructible, chewable and odorous) known to stimulate pigs’ interest [10]. Compared to the Rooting Cones, the Wood was manipulated less frequently, but for a longer time, giving it a longer mean manipulation length. Furthermore, the wood beams were presented directly on the floor and were not washed during experimentations. At the end of the five-day periods, they were soiled (personal observation), which is often associated with a decrease in interest [15,16]. However, the fact that the Wood was attached to a 1.5-m chain to limit its movements could have limited soiling and sustained the pigs’ interest.

The Ball triggered the lowest manipulation duration of all objects. Although the manipulation frequency was relatively high, the mean manipulation length of the Ball was the shortest of all objects. This finding is in accordance with results discussed in a review by Bracke et al. [11], showing low levels of manipulation toward hard plastic balls. This can be explained by the incapacity of the pigs to chew or to simulate a rooting behavior on them. In addition, the Ball was the most soiled object after five days (personal observation), potentially decreasing its attractiveness [16]. The Rooting Cones were also soiled, but less than the Ball, since they could not be moved around the pen. Although immobile objects on the ground are sometimes seen as unfavorable to stimulate manipulations [15], it has been found that an object can be manipulated for a longer time and in a more vigorous way when presented in a fixed montage rather than on a free-moving montage (e.g., Courboulay [20], Elkmann and Hoy [21]). The fixed presentation of the Rooting Cones may have allowed the pigs to express their rooting and chewing behaviors more satisfyingly, hence the high frequency and time spent manipulating them (Figure 2a,b). The pigs could also manipulate the Rooting Cones while lying on the floor, giving them the opportunity to express natural behaviors when resting. The pigs could use the area where the fixed Rooting Cones were located as a resting area, increasing manipulation time and the cleanliness of the area and the objects themselves [20].

Using a meta-analysis, Averós et al. [7] showed that one of the characteristics that had a significant impact on how much time the pigs spent manipulating objects is if they are suspended. Objects presented that way are more visible and less susceptible to being soiled [15]. The suspended objects in the present study (Bite-Rite, Disc, Porcichew and Seesaw) resulted in different manipulation durations and frequencies. However, the mean manipulation lengths were similar between the Bite-Rite, the Disc and the Porcichew, although greater for the Seesaw. The greater length of time spent on an object for each individual manipulation observed from the Seesaw may be partly related to the destructive and deformable nature of its suspended ropes [10]. The knots were untied, and the ropes were mostly destructed within the five-day periods. Such a progressive destruction of an object could sustain pigs’ interest in an object [22].

The Bite-Rite, Disc and Porcichew were subject to similar levels of manipulation. Low manipulation levels for the Bite-Rite were unexpected since the chewing sticks were deformable and destructible—two characteristics that seemed valued by pigs [10]. Chewing is one of the highly prioritized behaviors of pigs and a lack of chewable material is seen as a possible cause for harmful oral manipulations between pigs when placed in barren environments [12].

The Disc could have been less attractive to the pigs because it was hard to chew. Furthermore, the suspended chains around the disc could have been unattractive to the pigs as metal objects generally offer low enrichment benefits [11]. Chains have been associated with shorter manipulation times compared to other chewable objects, such as rubber hoses or ropes (e.g., Apple and Craig [23], Hill et al. [24]). It is also possible that the pigs were already accustomed to chains because all the pens had two suspended chains before experimentations began. The Porcichew was chewed more than the Disc, even though both objects were made of a similar hard plastic. The apple scent from the Porcichew could have helped to increase manipulations since odors are appealing to pigs [10].

In this research, analysis based on duration and mean manipulation length seemed to be more representative of the interest in the enrichment objects than the frequency of manipulation, according to the five-day manipulation patterns seen in Figure 1. This is in line with the proposition of Bracke [16] that an analysis solely based on parameters related to frequency could be insufficient to properly evaluate the welfare value of an enrichment object. For example, Telkänranta et al. [12] obtained similar manipulation frequencies for two enrichment objects, although only one of them reduced tail- and ear-biting in finishing pigs. The results from the present study offer different interpretations depending on how behavior was measured. Based on the frequency, the Rooting Cones would be the preferred enrichment object but based on time, the Wood would be due to both the duration and mean length of manipulation. However, the duration and mean length of manipulation with the Wood did not decrease over time, whereas these variables decreased significantly over time for the Rooting Cones, which suggests that the Wood may be a better option. Furthermore, the Ball and the Wood showed similar average frequencies of manipulation over five days, but the pigs spent over eight times as many seconds manipulating the Wood per manipulation. In addition, duration of manipulation changed differently over time between objects. The combination of both duration and frequency analyses, done as the mean manipulation length in this study, provides a more comprehensive explanation of the effects of enrichment objects on pigs’ interest.

### 4.2. Behavioral Analysis—Trial Two

The main finding of this experiment is the absence of a clear effect of washing or replacing an object daily on the behavior of pigs over five days. The only preferences detected were an increased frequency of contact with the unwashed Ball on day 2 and a longer mean manipulation length on day 4 with the unwashed Rooting Cones. The preference for the unwashed Ball did not translate into an increased manipulation duration. This may be explained by the general low level of attraction for a hard-plastic ball [11], as was also observed in trial one. The more frequent manipulations with the control Ball and Rooting Cones could be explained by the curiosity of the pigs toward the smell of the dirty object. Odor is considered as a potential characteristic enhancing interest in the objects [10] but in this case, the smell of the dirty object could have stimulated the pigs to initiate small bouts of manipulation while they were smelling them, which does not necessarily translate into an indication of high interest (as discussed in the previous Section 4.1). It is also possible that the odor from handling by the people who were cleaning the objects could have triggered some manipulations on both treatment and control objects. However, no measurements were taken in the hour following the reintroduction of the object in the pen. Therefore, it is not possible to quantify this potential effect.

This trial showed that a daily wash or replacement of the selected objects was unnecessary in the short term, based on the preference test. However, it could be necessary to wash or replace some objects after a longer period. For example, the Rope would be destroyed to a point where it would be unusable after a long period (control Rope was not too heavily destroyed after five days but it is estimated that another 10–15 days of manipulations would have resulted in complete destruction). In the study by Trickett et al. [17], in which the pigs had continuous access to ropes, replacement was needed every two weeks (14 days) due to progressive destruction. In our study, it was noted that the Ball was completely covered in dirt and feces by the fifth day in general. Longer-term studies would be necessary to determine the potential negative effect of a dirty object on pigs’ interest. Effects on pig health should also be measured, since dirty objects may represent a biosecurity risk [3].

The objects on the floor (Ball and Rooting Cones) were more affected by the decline of interest over time. These results are similar to those gathered in trial one and indicate that even when kept clean, those objects may not sustain pigs’ interest sufficiently over time. The Bite-Rite also provided results similar to those of trial one. The degradation of the control Rope had no effect on pigs’ interest, which as previously stated, could have been helpful to attract the pigs over time [22].

## 5. Conclusions

The level of pigs’ interest in enrichment objects was dependent on the characteristics and the presentation of the objects. The interest seemed greater toward the destructible and chewable objects, but only the piece of wood sustained pigs’ interest for the duration of the five-day period. Providing objects to growing pigs that have chewable and destructible properties could enhance their interest in them since they are capable of triggering species-specific behaviors and increasing the potential to reduce harmful contacts between pigs. However, those potential effects on pigs’ welfare should be tested in a long-term trial. In addition to pigs’ behavioral fulfillment, labor associated with the daily replacement or cleaning of objects can be minimized since it did not have a positive effect on their attractiveness. The characteristics of an enrichment object and the associated maintenance are especially important to optimize the impact of enrichment objects on welfare, while minimizing the associated labor cost.

## Figures and Tables

**Table 1:** Adjusted mean differences^1^ between treated (cleaned Ball, Bite-Rite and Rooting Cones or replaced Rope) and control (untouched) objects for the frequency of manipulations (F), duration of manipulation in seconds (D) and mean manipulation length in seconds (ML) from day 1 to 5, when both objects were presented simultaneously to growing pigs.

Object	Ball			Bite-Rite			Rope			Rooting Cones		
	F	D	ML	F	D	ML	F	D	ML	F	D	ML
**Day 1**	−2.25	179.6	10.45	−3.00	−132.6	−4.61	8.63	13.0	−17.22	5.33	24.6	−6.92
**Day 2**	−5.50 *	19.6	4.85	0.33	−107.3	−8.59	3.08	−175.8	−26.99	1.17	57.2	4.94
**Day 3**	−1.75	103.1	6.42	−2.33	−285.1	−18.32	3.92	130.6	3.80	−2.58	−220.4	−6.18 **
**Day 4**	−0.83	−7.9	−0.63	−1.83	−290.1	−20.57	0.83	391.3	32.32	3.00	−99.2	−8.48
**Day 5**	−1.17	−27.3	−2.63	−2.67	−161.0	−1.02	4.75	96.8	−0.29	1.08	4.5	−0.34

^1^ Negative differences (deltas) indicate more manipulations with control objects. * Indicates a significant preference for an object at *p* < 0.05, ** at *p* < 0.01 (pair-wise Student’s *t*-test).

## References

[1] Velarde A., Fàbrega E., Blanco-Penedo I., Dalmau A. (2015). Animal welfare towards sustainability in pork meat production. Meat Sci..

[2] EFSA (2007). Scientific report on the risks associated with tail biting in pigs and possible means to reduce the need for tail docking considering the different housing and husbandry systems. Efsa J..

[3] Van de Weerd H.A., Day J.E.L. (2009). A review of environmental enrichment for pigs housed in intensive housing systems. Appl. Anim. Behav. Sci..

[4] Broom D.M. (1998). Welfare, stress, and the evolution of feelings. Adv. Stud. Behav..

[5] Taylor N.R., Main D.C., Mendl M., Edwards S.A. (2010). Tail-biting: A new perspective. Vet. J..

[6] Sinisalo A., Niemi J.K., Heinonen M., Valros A. (2012). Tail biting and production performance in fattening pigs. Livestig. Sci..

[7] Averós X., Brossard L., Dourmad J.-Y., de Greef K.H., Edge H.L., Edwards S.A., Meunier-Salaün M.-C. (2010). A meta-analysis of the combined effect of housing and environmental enrichment characteristics on the behaviour and performance of pigs. Appl. Anim. Behav. Sci..

[8] Moinard C., Mendl M., Nicol C.J., Green L.E. (2003). A case control study of on-farm risk factors for tail biting in pigs. Appl. Anim. Behav. Sci..

[9] Day J.E.L., van de Weerd H.A., Edwards S.A. (2008). The effect of varying lengths of straw bedding on the behaviour of growing pigs. Appl. Anim. Behav. Sci..

[10] Van de Weerd H.A., Docking C.M., Day J.E.L., Avery P.J., Edwards S.A. (2003). A systematic approach towards developing environmental enrichment for pigs. Appl. Anim. Behav. Sci..

[11] Bracke M.B.M., Zonderland J.J., Lenskens P., Schouten W.G.P., Vermeer H., Spoolder H.A.M., Hendriks H.J.M., Hopster H. (2006). Formalised review of environmental enrichment for pigs in relation to political decision making. Appl. Anim. Behav. Sci..

[12] Telkänranta H., Bracke M.B.M., Valros A. (2014). Fresh wood reduces tail and ear biting and increases exploratory behaviour in finishing pigs. Appl. Anim. Behav. Sci..

[13] Zonderland J.J., Vermeer H.M., Vereijken P.F.G., Spoolder H.A.M. (2003). Measuring a pig’s preference for suspended toys by using an automated recording technique. CIGR Ej..

[14] Guy J.H., Meads Z.A., Shiel R.S., Edwards S.A. (2013). The effect of combining different environmental enrichment materials on enrichment use by growing pigs. Appl. Anim. Behav. Sci..

[15] Blackshaw J., Thomas F., Lee J. (1997). The effect of a fixed or free toy on the growth rate and aggressive behaviour of weaned pigs and the influence of hierarchy on initial investigation of the toys. Appl. Anim. Behav. Sci..

[16] Bracke M.B.M. (2007). Multifactorial testing of enrichment criteria: Pigs ‘demand’ hygiene and destructibility more than sound. Appl. Anim. Behav. Sci..

[17] Trickett S.L., Guy J.H., Edwards S.A. (2009). The role of novelty in environmental enrichment for the weaned pig. Appl. Anim. Behav. Sci..

[18] CCAC (2009). CCAC Guidelines on: The Care and Use of Farm Animals in Research, Teaching and Testing.

[19] Gifford A.K., Cloutier S., Newberry R.C. (2007). Objects as enrichment: Effects of object exposure time and delay interval on object recognition memory of the domestic pig. Appl. Anim. Behav. Sci..

[20] Courboulay V. (2004). Comment l’apport d’objets manipulables en hauteur et au sol influence-t-il l’activité des porcs charcutiers logés sur caillebotis intégral. J. Rech. Porc..

[21] Elkmann A., Hoy S. (2009). Frequency of occupation with different simultaneously offered devices by fattening pigs kept in pens with or without straw. Livest. Sci..

[22] Feddes J., Fraser D. (1994). Non-nutritive chewing by pigs: Implications for tail-biting and behavioral enrichment. Trans. Am. Soc. Agric. Eng..

[23] Apple J.K., Craig J.V. (1992). The influence of pen size on toy preference of growing pigs. Appl. Anim. Behav. Sci..

[24] Hill J.D., McGlone J.J., Fullwood S.D., Miller M.F. (1998). Environmental enrichment influences on pig behavior, performance and meat quality. Appl. Anim. Behav. Sci..

